# Cervical cytology and human papillomavirus among asymptomatic healthy volunteers in Vientiane, Lao PDR

**DOI:** 10.1186/s12885-017-3900-6

**Published:** 2017-12-19

**Authors:** Reika Takamatsu, Viengvansay Nabandith, Vatsana Pholsena, Phouthasone Mounthisone, Katsu Nakasone, Kentarou Ohtake, Naoki Yoshimi

**Affiliations:** 1 Department of Pathology and Oncology, Graduate School of Medicine, University of the Ryukyus, 207 Uehara, Nishihara-cho, Nakagami-gun, Okinawa, 903-0215 Japan; 2 Setthathirath Hospital, Donekoi Village, Sisatthanak District, P.O.Box 527, Vientiane, Lao PDR; 3 Mittaphab Hospital, Phonsavang Village, Chanthabouly District, P.O.Box 6035, Vientiane, Lao PDR; 4 Department of Pathology, Ryukyu University Hospital, 207 Uehara, Nishihara-cho, Nakagami-gun, Okinawa, 903-0215 Japan; 5 Clinical Laboratory, Medical Examination Center of Chubu Medical Association, 1-584 Miyagi, Chatan-cho, Nakagami-gun, Okinawa, 904-0113 Japan

**Keywords:** Human papillomavirus (HPV), Cytology, HPV testing, Vientiane

## Abstract

**Background:**

Cervical cancer is the most common cancer in women living in Vientiane, Lao People’s Democratic Republic (PDR). This study examines cervical cytology using a liquid-based cytology (LBC) method and reports the presence of high-risk (HR) human papillomavirus (HPV).

**Methods:**

We collected cervical samples from 1475 asymptomatic and healthy volunteers from six hospitals in Lao PDR. A total of 1422 volunteers (mean age 39.1 ± 6.4 years, range 30-54 years) were included in the final analysis. We performed HPV typing using the polymerase chain reaction technique to detect HR-HPV samples with abnormal cytology.

**Results:**

The overall rates of abnormal cytology and HR-HPV–positive in the samples were 9.3% (132/1422) and 47.7% (63/132), respectively. The samples with abnormal cytology included 13 high-grade squamous intraepithelial lesions and one squamous cell carcinoma case. The results showed that the most common type of HPV was HPV16 (20.5%) followed by HPV58 (9.1%).

**Conclusions:**

Healthy women in Vientiane, the capital of Lao PDR, have high rates of abnormal cervical cytology and are likely to be HR-HPV-positive. A system for detection and prevention of cervical cancer in these women should be developed in the near future.

**Electronic supplementary material:**

The online version of this article (10.1186/s12885-017-3900-6) contains supplementary material, which is available to authorized users.

## Background

Cervical cancer is the fourth most common cancer in women worldwide [1]. Although the incidence and mortality of cervical cancer have declined substantially in developed countries over the last four decades, more than 80% of cases occur in developing countries [2]. The disproportionate burden is considered to be due to the absence of well-organized cancer screening programs [2, 3]. In many developing countries, screening for cervical cancer is similar to what was done in the United States until the 1960s**,** when cervical cytology (the Papanicolaou [PAP] test) began to be used to detect cervical cancer [4]. Unfortunately, many developing countries, including Lao People’s Democratic Republic (Lao PDR), lack healthcare resources and funds for cytology screening programs [2].

Lao PDR is a landlocked country with a population of 6.37 million in Southeast Asia; the capital is Vientiane. This nation has been developing at a rapid pace. Cervical cancer was estimated to be the most common cancer among women in Lao PDR in 2010 [5], but 2012 findings showed it was the third most common cancer, following hepatocarcinoma and breast cancer [2].

Human papillomavirus (HPV), the most common sexually transmitted infection (STI) worldwide [6], has been strongly associated with cervical cancer. HPV is classified as low-risk or high-risk (HR) according to its role in the development of cervical cancer [7, 8]. At least 13 genotypes of HR-HPV (16, 18, 31, 33, 35, 39, 45, 51, 52, 56, 58, 59, and 68) have been found to be associated with the risk for developing cervical cancer [9]. HPV is classified as an STI, and it is important to determine the presence of an infection in women. It has recently been noted that HPV testing is being performed as part of primary cervical cancer screening in the United States and European countries such as England and the Netherlands [10, 11]. Phongsavan et al. reported the HPV infection rate in 1922 women from three provinces in Lao PDR [12]. According to their findings, 11% of women were positive for HR-HPV, but none had undergone cytological testing.

We previously reported on a cervical cytology screening program for healthy Lao women aged 30 years and older using a self-collecting instrument in cooperation with Laotian pathologists and Japanese pathologists in 2007-2010 [13, 14]. In this current report, we used Kato’s device and liquid-based cytology (LBC) to identify HPV genotypes and perform cytological examinations in healthy women in Lao PDR.

## Methods

**Fig. 1:**
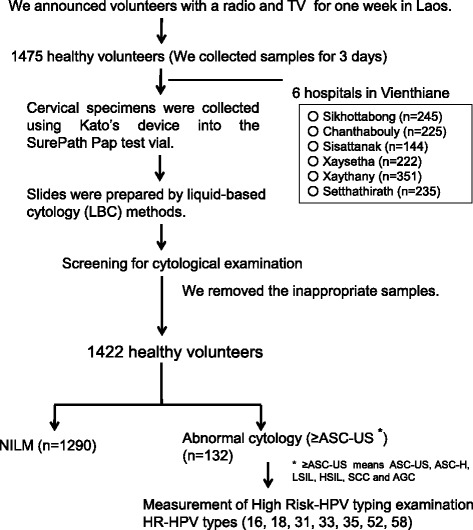
Study design

The study design is shown in Fig. 1. The study was conducted in December 2012. It received approval from the Ethics Committee for Clinical Research of the University of the Ryukyus and the National Ethics Committee of the Ministry of Health of Lao PDR. This study was similar in design to our previous trials [13, 14]. In brief, we used radio and television advertisements once a day for 1 week to announce that we were looking for volunteers. Asymptomatic women (1475 subjects) older than 30 years participated over 3 days at six hospitals (Setthathirath, Sikhottabong, Xaysetha, Chanthabouly, Sisattanak, Saythany), which are located in the capital city of Vientiane and the surrounding region. The volunteer women were explained the study and instructed how to use Kato’s device (Uterus Cancer Examination Instrument Manufactory, Nagoya) and to obtain the self-collected smear, by Lao doctors (V. Pholsena and V. Nabandith) or nurses, as was done in previous studies [13, 14], then provided written informed consent. We used questionnaires to gather information on age, marital status, number of children, and educational background. Questionnaires were originally written in the Lao language (Additional file 1), but nurses interviewed volunteers orally, because some participants were illiterate.

Cervical specimens collected using Kato’s device [15] were placed into SurePath Pap test vials and slides were prepared by LBC methods (SurePath LBC, Beckton Dickinson, Franklin Lakes, NJ). We confirmed cervical pathology based on the Bethesda system.

DNA was extracted using the phenol-chloroform protocol. The target HR-HPV DNA (consisting of seven types of HR-HPV: types 16, 18, 31, 33, 35, 52, and 58) was amplified by polymerase chain reaction (PCR) using TaKaRa PCR Human Papillomavirus Typing Set (Takara Bio Inc., Shiga, Japan), according to the manufacturer’s protocol. The PCR products were confirmed using agarose gels. Restriction enzyme digestion of PCR products can also be confirmed because PCR products recognize the sites of *Ava* II, *Afa* I, *Bgl* II, *Acc* I, and *Ava* I, and small DNA fragments are yielded by digestion of restriction enzymes. The DNA fragments were confirmed on agarose gels, and the HPV genotypes were identified.

All data were analyzed using GraphPad Prism (version 6, GraphPad Software, San Diego, CA). Statistical significance was defined as *P* < 0.05.

## Results

We collected and examined specimens from 1475 volunteers; due to inadequate specimens, our analysis was limited to 1422 women (mean age 39.1 ± 6.4 years, range 30-54 years) (Fig. 1).

### Cytological examination

**Fig. 2:**
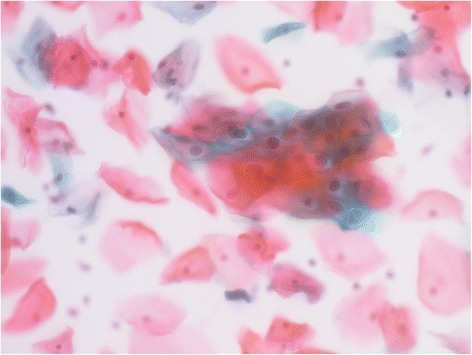
Representative cytological findings with LSIL in self-collected LBC specimens (PAP stains, ×40)

Self-collected LBC specimens had almost similar cytological features as the same conventional smears collected by gynecologists (Fig. 2). Cytological results based on the Bethesda system were classified as follows: negative for intraepithelial lesions or malignancy (NILM), 90.7% (1290/1422); atypical squamous cells of undetermined significance (ASC-US), 5.3% (75/1422); atypical squamous cells, cannot exclude high-grade squamous intraepithelial lesions (ASC-H), 0.6% (9/1422); low-grade squamous intraepithelial lesion (LSIL), 2.3% (33/1422), high-grade squamous intraepithelial lesions (HSIL), 0.9% (13/1422); atypical glandular cells (AGC), 0.07% (1/1422); and squamous cell carcinoma (SCC), 0.07% (1/1422). The overall prevalence of abnormal cytology (≥ASC-US, which means ASC-US, ASC-H, LSIL, HSIL, SCC and AGC), was 9.3% (132/1422). Findings of ≥ASC-US were not significantly associated with age, marital status, children, or education level, although HSIL trends showed an increased appearance in women with two or more children and were lower in women with higher education levels (Table 1).

**Table 1:** Summary of the characteristics of volunteers and the cytological results in this study

									number (%)
	n	NILM	≥ASC-US^a^	ASC-US	ASC-H	LSIL	HSIL	SCC	AGC
[Age]									
39.1 ± 6.4 (range 30-54)	1422	1290 (90.7)	132 (9.3)	75 (5.3)	9 (0.6)	33 (2.3)	13 (0.9)	1 (0.1)	1 (0.1)
30-34	414	380 (26.7)	34 (2.4)	16 (1.1)	1 (0.1)	15 (1.1)	2 (0.1)	0 (0.0)	0 (0.0)
35-39	320	290 (20.4)	30 (2.1)	16 (1.1)	3 (0.2)	5 (0.4)	5 (0.4)	0 (0.0)	1 (0.1)
40-44	319	287 (20.2)	32 (2.3)	20 (1.4)	1 (0.1)	8 (0.6)	3 (0.2)	0 (0.0)	0 (0.0)
45≦	369	333 (23.4)	36 (2.5)	23 (1.6)	4 (0.3)	5 (0.4)	3 (0.2)	1 (0.1)	0 (0.0)
[Marital status]									
Single	35	32 (2.3)	3 (0.2)	2 (0.1)	0 (0.0)	1 (0.1)	0 (0.0)	0 (0.0)	0 (0.0)
Married	1319	1197 (84.2)	122 (8.6)	67 (4.7)	9 (0.6)	31 (2.2)	13 (0.9)	1 (0.1)	1 (0.1)
unknown	68	61 (4.3)	7 (0.5)	6 (8.0)	0 (0.0)	1 (3.0)	0 (0.0)	0 (0.0)	0 (0.0)
[Number of children]									
0	70	66 (4.6)	4 (0.3)	1 (0.1)	0 (0.0)	3 (0.2)	0 (0.0)	0 (0.0)	0 (0.0)
1	218	198 (13.9)	20 (1.4)	11 (0.8)	1 (0.1)	6 (0.4)	2 (0.1)	0 (0.0)	0 (0.0)
2	572	521 (36.6)	51 (3.6)	26 (1.8)	5 (0.4)	14 (1.0)	5 (0.4)	0 (0.0)	1 (0.1)
3≦	438	389 (27.4)	49 (3.4)	32 (2.3)	3 (0.2)	7 (0.5)	6 (0.4)	1 (0.1)	0 (0.0)
unknown	124	116 (8.2)	8 (0.6)	5 (0.4)	0 (0.0)	3 (0.2)	0 (0.0)	0 (0.0)	0 (0.0)
[Education]									
No school	23	21 (1.5)	2 (0.1)	2 (0.1)	0 (0.0)	0 (0.0)	0 (0.0)	0 (0.0)	0 (0.0)
Primary	263	235 (16.5)	28 (2.0)	17 (1.2)	1 (0.1)	6 (0.4)	3 (0.2)	0 (0.0)	1 (0.1)
Secondary	5	0 (0.0)	5 (0.4)	0 (0.0)	5 (0.4)	0 (0.0)	0 (0.0)	0 (0.0)	0 (0.0)
High	391	352 (24.8)	39 (2.7)	24 (1.7)	3 (0.2)	7 (0.5)	5 (0.4)	0 (0.0)	0 (0.0)
College or university	280	266 (18.7)	14 (0.6)	12 (0.8)	0 (0.0)	8 (0.6)	0 (0.0)	0 (0.0)	0 (0.0)
unknown	439	416 (29.3)	23 (1.6)	5 (0.4)	0 (0.0)	12 (0.8)	5 (0.4)	1 (0.1)	0 (0.0)

^a^≥ ASC-US means ASC-US, ASC-H, LSIL, HSIL, SCC and AGC

### HPV analysis based on cytology

**Fig. 3:**
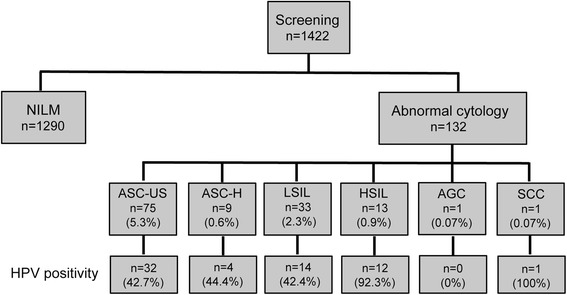
Summary of cytology and HPV infection

**Fig. 4:**
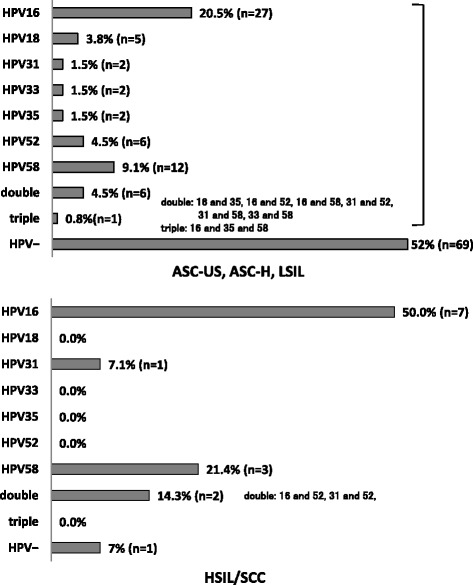
Relationship between cytology and HR-HPV infection. Significant difference between each group and HSIL/SCC (*P* < 0.01 by Fisher’s exact test).

**Fig. 5:**
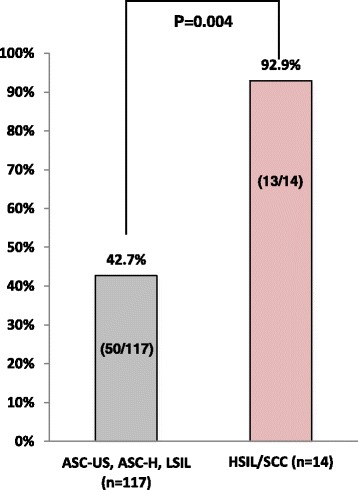
Distribution of HPV types in samples of both ≥ASC-US and HSIL/SCC

Figure 3 shows the incidence of HR-HPV–positive findings and includes 42.7% (32/75) of samples with ASC-US, 44.4% (4/9) with ASC-H, 42.4% (14/33) with LSIL, 92.3% (12/13) with HSIL, and 100% (1/1) with SCC. Figure 4 shows HR-HPV subtypes ≥ASC-US, and HSIL/SCC. The most prevalent HR-HPV types in cases with findings ≥ASC-US were HPV16, 20.5% (27/132); HPV58, 9.1% (12/132); double infection, 4.5% (6/132), including HPV16/35, HPV16/52, HPV16/58, HPV31/52, HPV31/58, and HPV33/58; and triple infection, 0.8% (1/132), including HPV16/35/58. The most prevalent HR-HPV types among women with HSIL/SCC were HPV16, 50% (7/14); HPV58, 21.4% (3/14); HPV31, 7.1% (1/14); and double infection, 14.3% (2/14), including HPV16/52 and HPV31/52 (Fig. 5).

## Discussion

According to a recent report by Sichanh et al. [16], most women in Lao PDR do not know that HPV causes cervical cancer. In this study, we first reported the relationship between cytology and HPV genotype in Lao PDR, which has not yet established a cervical cancer prevention screening system. Previously, we reported on cervical cytology of healthy women in Vientiane in 2008 and 2010; cytological findings included PAP ≥ Class IIIa, which includes IIIa, IIIb, IV and V in the PAP classification system, in 3% of 200 volunteers in 2008 [12] and ≥ASC-US in 5.7% of 1000 volunteers in 2010 [14]. However, the present rate of cytologic findings ≥ASC-US was 9.3% in 1422 volunteers, which was higher than that seen in our previous trials. The reason for the higher rate may be due to the use of the LBC procedure after specimen collection with Kato’s device. In fact, several reports have shown better performance with LBC compared with conventional PAP method [17, 18]. A cervical cancer prevention screening program in Okinawa, Japan, showed abnormal findings in 3.3% by LBC method in 2013 and 2014 but only 1.8% by a conventional PAP method in 2011 and 2012 [19]. Furthermore, when the PAP detection rates for cervical cancer in neighboring countries were observed, the results showed abnormal findings with a conventional PAP method were 4.9% in Thailand and 3.9% in Vietnam [20, 21]. Those data are similar to our previous findings using a conventional PAP method [13]. Meanwhile, the detection rate was reported 8.0% in Myanmar [22] when using a conventional PAP method, which is much higher than findings seen in this study in Lao PDR. Findings represented cytology of ≥ASC-US in 40% of cases in this study. In cases with ≥ASC-US, the positive rate of HR-HPV was 47.7% (63/132) and 92.9% (13/14) in HSIL and SCC cases, respectively. Cibas et al. also reported that the HR-HPV–positive rate for ASC-US ranged from 31.5% to 54.6% in abnormal cases [23]. In addition, there appear to be some distribution differences of HPV genotypes among different regions such as Africa, Asia, Europe, and the Americas [24, 25]. HPV genotypes 16, 18, 31, 52, and 58 are consistently found among the 10 most common types in all areas [24, 25]. Genotypes 16 and 18 have been considered to cause 70% of cervical cancers and precancerous cervical lesions [26]. Therefore, a 9-valent vaccine was recently developed in addition to a 2- or 4-valent vaccine for cervical cancer prevention [27]. In this study, HPV examination by PCR technique identified seven important genotypes: 16 in 20.5% of findings, 58 in 9.1%, 52 in 4.5%, 18 in 3.8%, and 31, 33, and 35 in 1.5% in participants with cytologic findings ≥ASC-US. Phongsavan et al. [10] also showed genotypes 33/52/58 in 4.3%, 16 in 3.1%, 18/45 in 1.5%, 56 in 1.4%, and 31 in 0.8% among their samples in Lao PDR. There seems to be high incidence of HPV58 in Lao PDR compared with other countries. Worldwide, it was reported that the most common HPV genotypes were 16, 18, and 52 [25]. Kantathavorn et al. reported that in Thailand, a neighboring country of Lao PDR, genotype 52 was the most detectable HPV, followed by genotype 16 [20]. In this study, we used a self-collecting device to collect cervical cytological material and did not perform histopathological examinations in volunteers with abnormal cytology and/or HR-HPV. We recognize that the accuracy of cytology collected by a gynecologist is better than that obtained through self-collection, and that it is necessary to do histopathological examinations for a precision management. However, Jeronimo et al. showed little difference in HPV test findings between clinician-collected and self-collected specimens [28]. In addition, cervical cytology is not popular, and limited resources lead to a lack of cytology screening programs in Lao PDR. In our previous studies [13, 14], we reported that 78% of participants preferred Kato’s device compared with collection by gynecologists. Yoshida et al. reported a similar preference by Lao women [29]. Furthermore, it is not common to examine the cervical smear in gynecological patients, as there are many gynecologists in Lao PDR, but they mainly work as obstetricians because of many births throughout the country. Therefore, the use of self-collecting device for cervical cytology may have applications in Lao PDR.

In addition, the use of social media such as radio and television to join us as volunteers may have been effective to study women in developing counties, because even a short advertisement could reach approximately 1500 participants. It seemed to be interested in cervical cancer for Lao women but they did not got used usual cytology because there are not specialists for cytology in Lao PDR. It means that it is necessary to built up the histopathology and cytology education system for medical staff in future.

## Conclusions

Healthy women in Vientiane, the capital of Lao PDR, have high rates of abnormal cervical cytology and are likely to be HR-HPV-positive. The most prevalent HR-HPV types in cases with findings ≥ASC-US were HPV16, 20.5%, HPV58, 9.1% and the double infection (16 and/or 58), 3.8%. Although it is desirable to perform LBC and HPV testing for cervical cancer screening, lack of resources makes this type of program unlikely in Lao PDR. Even when using the cheaper conventional PAP technique, there are some problems that can occur due to poor cytological technique as well as a lack of cytopathologists and cytotechnicians. These findings indicate that it is necessary to built a histopathology and cytology education system for medical staff in the future. In the meantime, we will continue PAP and HPV tests in Lao PDR using a self-collection procedure.

## Additional file

Additional file 1: Questionnaires in English. We asked the social status and knowledge about cervical cancer as questionnaires. It was originally written in the Lao language and this was translated in English. (PDF 248 kb)

## Abbreviations

AGC: Atypical glandular cells
ASC-H: Atypical squamous cells, cannot exclude high-grade squamous intraepithelial lesions
ASC-US: Atypical squamous cells of undetermined significance
HPV: Human papillomavirus
HR: High-risk
HSIL: High-grade squamous intraepithelial lesions
LBC: liquid-based cytology
LSIL: Low-grade squamous intraepithelial lesion
NLIM: Negative for intraepithelial lesions or malignancy
PAP: Papanicolaou
PCR: Polymerase chain reaction
PDR: People’s Democratic
SCC: Squamous cell carcinoma
STI: Sexually transmitted infection
