# Supplementary material for: Cervical cytology and human papillomavirus among asymptomatic healthy volunteers in Vientiane, Lao PDR
Source: BMC Cancer. 2017 Dec 19;17:872. doi: 10.1186/s12885-017-3900-6 (PMC5738160; doi:10.1186/s12885-017-3900-6)
Supplement: Additional file 1: — Questionnaires in English. We asked the social status and knowledge about cervical cancer as questionnaires. It was originally written in the Lao language and this was translated in English. (PDF 248 kb) [file 12885_2017_3900_MOESM1_ESM.pdf]

Questionnaires for Cervical Cancer Screening by using Self Sampling Device and HPV subtype examination in Lao PDR

I. Name and Family Name:..Age:..............Years old

II. Address; Village: District: Province:

III. What is your occupation?

①.House wife ☐ ②.Farmer ☐ ③.Government Employee ☐

④.Private Employee☐ ⑤.Business owner ☐

⑥.Other (Specify:..).

IV. Marital Status (Check in the parenthesis)

①.Single ☐ ②.Married ☐ ③ widowed ☐ ④.Devoice ☐

④.Have you ever gotten married before?.

Yes ☐ No ☐

V. Educational Background

①. No school ☐ ②. Primary School ☐ ③.Upper Secondary School ☐

④.High School ☐ ⑤.College or University ☐

VI. About Pregnancy.

How many time did your pregnant?.

①.Pregnancy:..............Time. ②.Don’t know ☐

③.How may children did you give birth?:............, ④.Never pregnant ☐

VII. About Cervical cancer.

Have your family member suffered with Cervical Cancer?.

①.Yes ☐ ②.No ☐

②.If Yes, who are they?.

Mother ☐ Older sister ☐ Younger sister ☐ Relative ☐

VIII. Knowledge about Cervical Cancer Screening.

1) Which one is the most effective of Cervical Cancer Screening?

①.Come to hospital to carry out Cervical cytology every 2 or 3 year and follow up in case presence of abnormal Cervical cells ☐

②.Go to see the doctor in case of Vaginal bleeding ☐

2) Which one is the best answer for The evaluation of Cervical cancer cells:

①.Cancer cells in Cervical cancer are different from Cancer cell in other system ☐

②. HPV is the most common cause of Cervical cell dysplasia and develop into Carcinoma ☐

3) What is Cervical Cytology examine? Which one is correct: (Check the Parenthesis)

①.Cytology is an examination to check the status of cell in human body ☐

②.Cytology cannot identify the Disease ☐

IX. Experience about Cervical Cancer Screening

Have you ever had a screening program for cervical cancer?

①.No ☐ ②.Yes ☐

If Yes:

②.1.How often do you have: ....Time; Don't know ☐

②.2.Where did you go for Cervical cancer screening?

Hospital ☐ Private Clinic ☐ Both Hospital and Private Clinic ☐

②.3.When do you usually go to see the doctor for Cervical cancer screening?

3 Months/Year ☐ 6 Months/Year ☐ Every Month ☐ Every Years ☐

②.4.How about the result?

Normal ☐ Inflammation ☐ Mild Dysplasia ☐ Carcinoma ☐

X. Expression about Cervical cancer screening program

①.How do you think about Cervical cancer screening by using self-sampling device?

Very good ☐ Good ☐ Not sure ☐ Not good ☐ Very bad ☐

XI. Which one do you prefer between Self sampling method and Gynecological examination?

①.I prefer self - sampling method ☐

②. I prefer Gynecological examination ☐

③.Don't know ☐

④.I prefer both ☐

XII.Other comment about Cervical cancer screening program
